# A four-armed Schiff base: 6,6′,6′′,6′′′-tetra­meth­oxy-2,2′,2′′,2′′′-[methanetetra­yltetra­kis(methylenenitrilo­methyl­idyne)]tetra­phenol

**DOI:** 10.1107/S1600536808020618

**Published:** 2008-07-12

**Authors:** Guang-Qi Jiang, Jie Cai, Yun-Qian Zhang, Qian-Jun Zhang

**Affiliations:** a Department of Chemistry, Guizhou University, Guiyang 550025, People’s Republic of China

## Abstract

In the structure of the title compound, C_37_H_40_N_4_O_8_, penta­erythrityltetra­mine is bonded to four *o*-vanillin mol­ecules, forming a four-armed Schiff base mol­ecule. These mol­ecules are connected by inter­molecular C—H⋯O hydrogen bonds. Intramolecular C—H⋯N and O—H⋯N hydrogen bonds are also present.

## Related literature

For related literature, see: Tanaka *et al.* (2006 ▶); Bernardo *et al.* (1996 ▶); Laliberte *et al.* (2004 ▶).
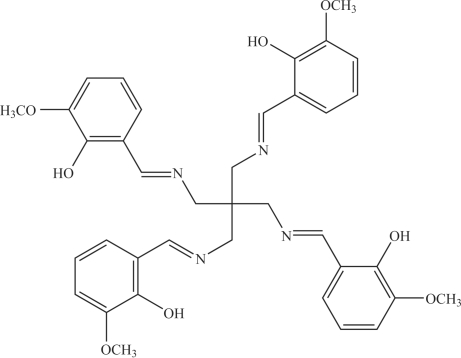


## Experimental

### 

#### Crystal data

C_37_H_40_N_4_O_8_
*M* *_r_* = 668.73
Triclinic, 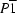

*a* = 11.3464 (11) Å
*b* = 12.4437 (12) Å
*c* = 13.0523 (14) Å
α = 75.861 (7)°
β = 88.893 (7)°
γ = 78.385 (7)°
*V* = 1749.6 (3) Å^3^
*Z* = 2
Mo *K*α radiation
μ = 0.09 mm^−1^
*T* = 273 (2) K
0.18 × 0.16 × 0.15 mm

#### Data collection

Bruker APEXII CCD area-detector diffractometer
Absorption correction: multi-scan (*SADABS*; Bruker, 2007 ▶) *T*_min_ = 0.984, *T*_max_ = 0.987
16510 measured reflections
6221 independent reflections
3077 reflections with *I* > 2σ(*I*)
*R*_int_ = 0.058

#### Refinement

*R*[*F* ^2^ > 2σ(*F* ^2^)] = 0.054
*wR*(*F* ^2^) = 0.145
*S* = 0.94
6194 reflections
443 parameters
H-atom parameters constrained
Δρ_max_ = 0.18 e Å^−3^
Δρ_min_ = −0.19 e Å^−3^

### 

Data collection: *APEX2* (Bruker, 2007 ▶); cell refinement: *SAINT* (Bruker, 2007 ▶); data reduction: *SAINT* (Bruker, 2007 ▶); program(s) used to solve structure: *SHELXS97* (Sheldrick, 2008 ▶); program(s) used to refine structure: *SHELXL97* (Sheldrick, 2008 ▶); molecular graphics: *ORTEP-3 for Windows* (Farrugia, 1997 ▶); software used to prepare material for publication: *WinGX* (Farrugia, 1999 ▶).

## Supplementary Material

Crystal structure: contains datablocks global, I. DOI: 10.1107/S1600536808020618/om2247sup1.cif

Structure factors: contains datablocks I. DOI: 10.1107/S1600536808020618/om2247Isup2.hkl

Additional supplementary materials:  crystallographic information; 3D view; checkCIF report

## Figures and Tables

**Table 1:** Hydrogen-bond geometry (Å, °)

*D*—H⋯*A*	*D*—H	H⋯*A*	*D*⋯*A*	*D*—H⋯*A*
O2—H2⋯N1	0.82	1.87	2.592 (3)	147
O4—H4⋯N4	0.82	1.84	2.567 (2)	147
O6—H6⋯N3	0.82	1.89	2.623 (3)	148
O7—H7⋯N2	0.82	1.88	2.612 (3)	148
C27—H27*B*⋯N1	0.97	2.56	2.936 (3)	103
C26—H26⋯O5^i^	0.93	2.58	3.309 (3)	136
C28—H28*C*⋯O2^i^	0.96	2.57	3.387 (3)	143
C12—H12⋯O6^ii^	0.93	2.59	3.400 (3)	146
C32—H32⋯O3^iii^	0.93	2.42	3.289 (3)	155
C36—H36*B*⋯O8^iv^	0.97	2.55	3.399 (3)	146

Symmetry codes: (i) 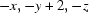; (ii) 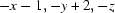; (iii) 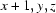; (iv) 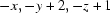.

**Fig. 1.**
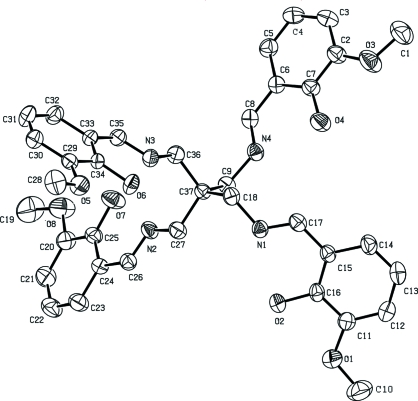
The molecular structure shown with 50% probability displacement ellipsoids.

**Fig. 2.**
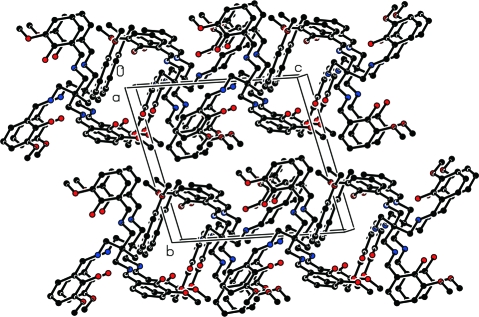
One-dimensional chains of the Schiff base perpendicular to the bc plane, showing the packing of the compound. Hydrogen bonds and C—H···π interactions are shown as dashed lines. H atoms have been omitted for clarity.

## supplementary crystallographic information

### Comment

Schiff bases have been intensively investigated during the past decades, mainly because of their strong coordination capability and diverse biological activities, such as antibacterial, antitumor activities, *etc.* (Tanaka *et al.*, 2006; Bernardo *et al.*, 1996). Pentaerythrityl tetramine and its congeners act as versatile building blocks in supramolecular chemistry and have been widely employed for the synthesis of various spiro crown ethers, spiro-macrocyclic ligands *etc.* (Laliberte *et al.*, 2004). However, to the best of our knowledge, few Schiff bases and their complexes derived from pentaerythrityl tetramine have been reported. Herein,we present the synthesis and crystal structure of the multidentate Schiff base ligand derived from *o*-vanillin and pentaerythrityl tetramine.

The molecular structure is shown in Fig.1. The atoms C9, C18, C27, and C36 form a distorted tetrahedral geometry about the central atom C37. The structure reveals four intramolecular O—H···N hydrogen bonds and one C—H···N hydrogen bond as well as five intermolecular C—H···O hydrogen bonds (Table 1). A view of the packing is given in Fig. 2. The molecules are also connected by C—H···π interactions, with C9—H9B···*Cg*(1) angle of 138 °, C9···*Cg*(1) distance of 3.595 (3) Å and C14—H14···*Cg*(4) angle of 157 °, C14···*Cg*(4) distance of 3.614 (3) Å, where *Cg*(1) and *Cg*(4) are the centroids of the phenyl ring formed by atoms C2,C3,C4,C5,C6,C7 and C29,C30,C31,C32,C33,C34 at (1 - *x*,-*y*,1 - *z*) and (1 + *x*,*y*,*z*), respectively.

### Experimental

A solution of pentaerythrityl tetramine (0.13 g, 1 mmol) in ethanol (20 ml) was added dropwise while stirring to a solution of *o*-vanillin (0.68 g, 4 mmol) in ethanol (20 ml),the mixture was stirred for about 2 h at room temperature. The resulting solution was filtered, and after slow evaporation of the solvent for several days, crystals suitable for X-ray diffraction were obtained in a yield of 46.4%.

### Refinement

All H atoms were placed in calculated positions and refined as riding, with C—H = 0.93–0.97 Å, and with *U*_iso_(H) = 1.2–1.5 *U*_eq_ (C,*O*).

### Crystal data

**Table:**

C_37_H_40_N_4_O_8_	*Z* = 2
*M**_r_* = 668.73	*F*_000_ = 708
Triclinic, *P*1	*D*_x_ = 1.269 Mg m^−^^3^
Hall symbol: -P 1	Mo *K*α radiation λ = 0.71073 Å
*a* = 11.3464 (11) Å	Cell parameters from 16510 reflections
*b* = 12.4437 (12) Å	θ = 1.0–25.1º
*c* = 13.0523 (14) Å	µ = 0.09 mm^−^^1^
α = 75.861 (7)º	*T* = 273 (2) K
β = 88.893 (7)º	Prism, yellow
γ = 78.385 (7)º	0.18 × 0.16 × 0.15 mm
*V* = 1749.6 (3) Å^3^	

### Data collection

**Table:**

Bruker APEXII CCD area-detector diffractometer	6221 independent reflections
Radiation source: fine-focus sealed tube	3077 reflections with *I* > 2σ(*I*)
Monochromator: graphite	*R*_int_ = 0.058
*T* = 273(2) K	θ_max_ = 25.1º
φ and ω scans	θ_min_ = 1.7º
Absorption correction: multi-scan(SADABS; Bruker, 2007)	*h* = −13→13
*T*_min_ = 0.984, *T*_max_ = 0.987	*k* = −14→14
16510 measured reflections	*l* = −13→15

### Refinement

**Table:**

Refinement on *F*^2^	Hydrogen site location: inferred from neighbouring sites
Least-squares matrix: full	H-atom parameters constrained
*R*[*F*^2^ > 2σ(*F*^2^)] = 0.054	*w* = 1/[σ^2^(*F*_o_^2^) + (0.0583*P*)^2^] where *P* = (*F*_o_^2^ + 2*F*_c_^2^)/3
*wR*(*F*^2^) = 0.145	(Δ/σ)_max_ < 0.001
*S* = 0.94	Δρ_max_ = 0.18 e Å^−^^3^
6194 reflections	Δρ_min_ = −0.19 e Å^−^^3^
443 parameters	Extinction correction: SHELXL97 (Sheldrick, 2008), Fc^*^=kFc[1+0.001xFc^2^λ^3^/sin(2θ)]^-1/4^
Primary atom site location: structure-invariant direct methods	Extinction coefficient: 0.0093 (14)
Secondary atom site location: difference Fourier map	

### Special details

**Table:**

Geometry. All e.s.d.'s (except the e.s.d. in the dihedral angle between two l.s. planes) are estimated using the full covariance matrix. The cell e.s.d.'s are taken into account individually in the estimation of e.s.d.'s in distances, angles and torsion angles; correlations between e.s.d.'s in cell parameters are only used when they are defined by crystal symmetry. An approximate (isotropic) treatment of cell e.s.d.'s is used for estimating e.s.d.'s involving l.s. planes.
Refinement. Refinement of *F*^2^ against ALL reflections. The weighted *R*-factor *wR* and goodness of fit *S* are based on *F*^2^, conventional *R*-factors *R* are based on *F*, with *F* set to zero for negative *F*^2^. The threshold expression of *F*^2^ > σ(*F*^2^) is used only for calculating *R*-factors(gt) *etc*. and is not relevant to the choice of reflections for refinement. *R*-factors based on *F*^2^ are statistically about twice as large as those based on *F*, and *R*- factors based on ALL data will be even larger.

### Fractional atomic coordinates and isotropic or equivalent isotropic displacement parameters (Å^2^)

**Table:**

	*x*	*y*	*z*	*U*_iso_*/*U*_eq_	
C1	−0.6783 (3)	0.6040 (3)	0.6346 (3)	0.1205 (14)	
H1A	−0.7350	0.5856	0.5914	0.181*	
H1B	−0.7208	0.6457	0.6816	0.181*	
H1C	−0.6285	0.5355	0.6752	0.181*	
C2	−0.5190 (3)	0.7057 (2)	0.6164 (2)	0.0661 (8)	
C3	−0.4819 (3)	0.6681 (2)	0.7207 (2)	0.0689 (8)	
H3	−0.5142	0.6112	0.7650	0.083*	
C4	−0.3970 (3)	0.7138 (2)	0.7606 (2)	0.0705 (8)	
H4A	−0.3729	0.6879	0.8315	0.085*	
C5	−0.3483 (2)	0.7971 (2)	0.6962 (2)	0.0637 (7)	
H5	−0.2922	0.8283	0.7239	0.076*	
C6	−0.3823 (2)	0.8355 (2)	0.5891 (2)	0.0504 (6)	
C7	−0.4681 (2)	0.7891 (2)	0.5489 (2)	0.0561 (7)	
C8	−0.3299 (2)	0.9225 (2)	0.5203 (2)	0.0556 (7)	
H8	−0.2759	0.9550	0.5492	0.067*	
C9	−0.2957 (2)	1.0390 (2)	0.35387 (19)	0.0541 (7)	
H9A	−0.2517	1.0717	0.3972	0.065*	
H9B	−0.3559	1.0994	0.3116	0.065*	
C10	−0.7806 (3)	1.3762 (3)	−0.1285 (3)	0.1135 (13)	
H10A	−0.7593	1.4442	−0.1701	0.170*	
H10B	−0.8450	1.3953	−0.0832	0.170*	
H10C	−0.8063	1.3352	−0.1746	0.170*	
C11	−0.6932 (2)	1.2070 (2)	−0.0018 (2)	0.0585 (7)	
C12	−0.7994 (2)	1.1700 (3)	0.0167 (2)	0.0672 (8)	
H12	−0.8709	1.2161	−0.0145	0.081*	
C13	−0.8005 (3)	1.0645 (3)	0.0816 (3)	0.0773 (9)	
H13	−0.8725	1.0393	0.0929	0.093*	
C14	−0.6962 (2)	0.9971 (2)	0.1292 (2)	0.0680 (8)	
H14	−0.6979	0.9259	0.1725	0.082*	
C15	−0.5875 (2)	1.0334 (2)	0.11382 (19)	0.0511 (7)	
C16	−0.5865 (2)	1.1396 (2)	0.0472 (2)	0.0527 (7)	
C17	−0.4787 (2)	0.9634 (2)	0.16885 (19)	0.0567 (7)	
H17	−0.4822	0.8927	0.2121	0.068*	
C18	−0.2728 (2)	0.9217 (2)	0.21916 (19)	0.0540 (7)	
H18A	−0.2174	0.8917	0.1707	0.065*	
H18B	−0.2971	0.8584	0.2683	0.065*	
C19	0.1639 (4)	1.4133 (3)	0.5082 (3)	0.1264 (14)	
H19A	0.1696	1.3906	0.5840	0.190*	
H19B	0.1277	1.4923	0.4858	0.190*	
H19C	0.2429	1.4004	0.4803	0.190*	
C20	0.0741 (2)	1.3692 (2)	0.3635 (2)	0.0667 (8)	
C21	0.1075 (3)	1.4562 (2)	0.2895 (3)	0.0770 (9)	
H21	0.1441	1.5075	0.3116	0.092*	
C22	0.0876 (3)	1.4686 (2)	0.1828 (3)	0.0822 (9)	
H22	0.1117	1.5272	0.1334	0.099*	
C23	0.0320 (2)	1.3938 (2)	0.1502 (2)	0.0727 (8)	
H23	0.0183	1.4023	0.0784	0.087*	
C24	−0.0044 (2)	1.3052 (2)	0.2232 (2)	0.0509 (7)	
C25	0.0163 (2)	1.2936 (2)	0.3301 (2)	0.0581 (7)	
C26	−0.0634 (2)	1.2277 (2)	0.1863 (2)	0.0547 (7)	
H26	−0.0732	1.2373	0.1138	0.066*	
C27	−0.1624 (2)	1.0776 (2)	0.19919 (19)	0.0591 (7)	
H27A	−0.1064	1.0425	0.1541	0.071*	
H27B	−0.2297	1.1260	0.1550	0.071*	
C28	0.3376 (3)	0.6069 (3)	−0.0230 (2)	0.0883 (10)	
H28A	0.3091	0.6053	−0.0912	0.132*	
H28B	0.3572	0.5312	0.0209	0.132*	
H28C	0.4082	0.6394	−0.0308	0.132*	
C29	0.2713 (2)	0.6854 (2)	0.1230 (2)	0.0519 (7)	
C30	0.3835 (2)	0.6521 (2)	0.1727 (2)	0.0666 (8)	
H30	0.4489	0.6186	0.1394	0.080*	
C31	0.3984 (3)	0.6687 (3)	0.2720 (3)	0.0782 (9)	
H31	0.4743	0.6462	0.3052	0.094*	
C32	0.3036 (2)	0.7176 (2)	0.3220 (2)	0.0675 (8)	
H32	0.3149	0.7272	0.3894	0.081*	
C33	0.1896 (2)	0.7534 (2)	0.27244 (19)	0.0493 (6)	
C34	0.1742 (2)	0.7371 (2)	0.1721 (2)	0.0472 (6)	
C35	0.0911 (2)	0.8099 (2)	0.3253 (2)	0.0543 (7)	
H35	0.1062	0.8195	0.3919	0.065*	
C36	−0.1053 (2)	0.9013 (2)	0.3478 (2)	0.0583 (7)	
H36A	−0.0667	0.9410	0.3885	0.070*	
H36B	−0.1378	0.8435	0.3973	0.070*	
C37	−0.2085 (2)	0.9849 (2)	0.28059 (18)	0.0475 (6)	
N1	−0.37817 (18)	0.99528 (17)	0.16006 (15)	0.0527 (5)	
N2	−0.10224 (18)	1.14746 (18)	0.24823 (16)	0.0586 (6)	
N3	−0.01463 (19)	0.84665 (17)	0.28493 (15)	0.0544 (6)	
N4	−0.35536 (17)	0.95591 (17)	0.42188 (17)	0.0542 (6)	
O1	−0.68132 (17)	1.30936 (18)	−0.06743 (17)	0.0877 (6)	
O2	−0.48359 (14)	1.17979 (14)	0.02791 (14)	0.0690 (5)	
H2	−0.4272	1.1331	0.0611	0.104*	
O3	−0.6060 (2)	0.6698 (2)	0.56997 (16)	0.1045 (8)	
O4	−0.50509 (17)	0.82276 (16)	0.44657 (14)	0.0791 (6)	
H4	−0.4688	0.8710	0.4150	0.119*	
O5	0.24695 (16)	0.67307 (15)	0.02443 (14)	0.0692 (5)	
O6	0.06588 (14)	0.77182 (15)	0.12026 (13)	0.0641 (5)	
H6	0.0164	0.8014	0.1570	0.096*	
O7	−0.01615 (18)	1.20882 (16)	0.40428 (14)	0.0870 (7)	
H7	−0.0487	1.1704	0.3754	0.131*	
O8	0.0914 (2)	1.34865 (17)	0.47009 (17)	0.1004 (7)	

### Atomic displacement parameters (Å^2^)

**Table:**

	*U*^11^	*U*^22^	*U*^33^	*U*^12^	*U*^13^	*U*^23^
C1	0.130 (3)	0.122 (3)	0.118 (3)	−0.077 (3)	0.002 (3)	−0.005 (3)
C2	0.080 (2)	0.0671 (19)	0.0570 (19)	−0.0297 (17)	0.0043 (16)	−0.0143 (16)
C3	0.091 (2)	0.0606 (19)	0.0545 (19)	−0.0213 (17)	0.0114 (17)	−0.0093 (16)
C4	0.087 (2)	0.071 (2)	0.0492 (17)	−0.0113 (17)	0.0006 (16)	−0.0090 (16)
C5	0.0650 (18)	0.076 (2)	0.0539 (18)	−0.0153 (16)	−0.0037 (14)	−0.0208 (16)
C6	0.0527 (15)	0.0560 (16)	0.0465 (16)	−0.0115 (13)	0.0051 (13)	−0.0200 (14)
C7	0.0656 (17)	0.0617 (18)	0.0441 (16)	−0.0180 (15)	0.0041 (14)	−0.0149 (14)
C8	0.0527 (16)	0.0623 (18)	0.0592 (18)	−0.0135 (14)	0.0050 (14)	−0.0273 (15)
C9	0.0607 (16)	0.0543 (16)	0.0512 (16)	−0.0170 (14)	0.0047 (14)	−0.0160 (14)
C10	0.117 (3)	0.082 (2)	0.121 (3)	−0.005 (2)	−0.033 (3)	0.005 (2)
C11	0.0613 (18)	0.0593 (19)	0.0583 (18)	−0.0161 (15)	−0.0014 (15)	−0.0173 (15)
C12	0.0532 (17)	0.076 (2)	0.075 (2)	−0.0103 (15)	−0.0054 (15)	−0.0256 (18)
C13	0.0562 (19)	0.088 (2)	0.097 (2)	−0.0291 (18)	0.0082 (18)	−0.030 (2)
C14	0.0591 (18)	0.0647 (19)	0.086 (2)	−0.0271 (16)	0.0036 (16)	−0.0172 (17)
C15	0.0527 (16)	0.0530 (16)	0.0543 (16)	−0.0208 (13)	0.0028 (13)	−0.0176 (14)
C16	0.0481 (16)	0.0660 (18)	0.0525 (16)	−0.0204 (14)	0.0039 (13)	−0.0236 (14)
C17	0.0643 (18)	0.0588 (17)	0.0540 (17)	−0.0225 (15)	0.0071 (14)	−0.0192 (14)
C18	0.0551 (16)	0.0551 (16)	0.0571 (16)	−0.0163 (14)	0.0036 (13)	−0.0197 (14)
C19	0.164 (4)	0.111 (3)	0.124 (3)	−0.049 (3)	−0.046 (3)	−0.046 (3)
C20	0.076 (2)	0.0602 (19)	0.067 (2)	−0.0202 (16)	−0.0088 (16)	−0.0167 (17)
C21	0.084 (2)	0.0555 (19)	0.097 (3)	−0.0239 (17)	−0.0023 (19)	−0.0208 (19)
C22	0.096 (2)	0.064 (2)	0.088 (3)	−0.0369 (18)	0.005 (2)	−0.0059 (19)
C23	0.084 (2)	0.070 (2)	0.0649 (19)	−0.0292 (17)	0.0038 (16)	−0.0058 (17)
C24	0.0545 (15)	0.0473 (16)	0.0500 (17)	−0.0126 (13)	0.0033 (13)	−0.0084 (13)
C25	0.0637 (17)	0.0510 (17)	0.0593 (18)	−0.0201 (14)	−0.0005 (14)	−0.0060 (15)
C26	0.0529 (16)	0.0597 (17)	0.0521 (16)	−0.0138 (14)	0.0036 (13)	−0.0132 (14)
C27	0.0687 (17)	0.0664 (17)	0.0486 (16)	−0.0295 (15)	−0.0004 (13)	−0.0130 (14)
C28	0.087 (2)	0.094 (2)	0.097 (2)	−0.0184 (19)	0.023 (2)	−0.050 (2)
C29	0.0546 (17)	0.0526 (16)	0.0486 (16)	−0.0155 (13)	0.0055 (14)	−0.0091 (13)
C30	0.0521 (18)	0.0663 (19)	0.076 (2)	−0.0059 (14)	0.0028 (16)	−0.0117 (17)
C31	0.0482 (18)	0.095 (2)	0.087 (2)	−0.0025 (16)	−0.0133 (17)	−0.021 (2)
C32	0.0580 (18)	0.084 (2)	0.0589 (18)	−0.0105 (16)	−0.0107 (15)	−0.0164 (16)
C33	0.0467 (15)	0.0541 (16)	0.0467 (15)	−0.0128 (13)	−0.0049 (13)	−0.0088 (13)
C34	0.0379 (14)	0.0488 (15)	0.0529 (16)	−0.0134 (12)	−0.0039 (13)	−0.0048 (13)
C35	0.0574 (17)	0.0608 (17)	0.0476 (16)	−0.0183 (14)	−0.0043 (14)	−0.0130 (14)
C36	0.0566 (16)	0.0681 (18)	0.0516 (16)	−0.0115 (14)	0.0032 (14)	−0.0183 (14)
C37	0.0524 (15)	0.0493 (15)	0.0433 (15)	−0.0156 (13)	0.0027 (13)	−0.0120 (13)
N1	0.0511 (13)	0.0596 (14)	0.0513 (13)	−0.0157 (11)	0.0025 (11)	−0.0175 (11)
N2	0.0651 (14)	0.0654 (15)	0.0520 (13)	−0.0311 (12)	0.0027 (11)	−0.0123 (12)
N3	0.0477 (13)	0.0636 (14)	0.0534 (13)	−0.0122 (11)	0.0016 (11)	−0.0162 (11)
N4	0.0533 (13)	0.0612 (14)	0.0519 (14)	−0.0156 (11)	0.0086 (11)	−0.0182 (12)
O1	0.0758 (14)	0.0767 (14)	0.0974 (16)	−0.0167 (12)	−0.0118 (12)	0.0055 (13)
O2	0.0564 (11)	0.0705 (12)	0.0794 (13)	−0.0261 (10)	0.0020 (10)	−0.0061 (10)
O3	0.1308 (19)	0.1312 (19)	0.0715 (14)	−0.0868 (17)	0.0009 (13)	−0.0134 (14)
O4	0.0976 (14)	0.0991 (15)	0.0501 (12)	−0.0504 (12)	−0.0052 (10)	−0.0104 (11)
O5	0.0691 (12)	0.0804 (14)	0.0596 (12)	−0.0096 (11)	0.0072 (10)	−0.0249 (11)
O6	0.0455 (10)	0.0858 (13)	0.0635 (11)	−0.0079 (9)	−0.0075 (9)	−0.0268 (10)
O7	0.1317 (18)	0.0847 (14)	0.0563 (12)	−0.0599 (14)	−0.0076 (12)	−0.0072 (11)
O8	0.149 (2)	0.0852 (15)	0.0793 (16)	−0.0509 (15)	−0.0278 (14)	−0.0184 (13)

### Geometric parameters (Å, °)

**Table:**

C1—O3	1.401 (3)	C19—H19B	0.9600
C1—H1A	0.9600	C19—H19C	0.9600
C1—H1B	0.9600	C20—O8	1.362 (3)
C1—H1C	0.9600	C20—C21	1.375 (3)
C2—O3	1.369 (3)	C20—C25	1.402 (4)
C2—C3	1.371 (3)	C21—C22	1.381 (4)
C2—C7	1.400 (3)	C21—H21	0.9300
C3—C4	1.381 (4)	C22—C23	1.373 (4)
C3—H3	0.9300	C22—H22	0.9300
C4—C5	1.369 (3)	C23—C24	1.396 (3)
C4—H4A	0.9300	C23—H23	0.9300
C5—C6	1.397 (3)	C24—C25	1.387 (3)
C5—H5	0.9300	C24—C26	1.453 (3)
C6—C7	1.397 (3)	C25—O7	1.353 (3)
C6—C8	1.447 (3)	C26—N2	1.270 (3)
C7—O4	1.346 (3)	C26—H26	0.9300
C8—N4	1.270 (3)	C27—N2	1.469 (3)
C8—H8	0.9300	C27—C37	1.535 (3)
C9—N4	1.457 (3)	C27—H27A	0.9700
C9—C37	1.535 (3)	C27—H27B	0.9700
C9—H9A	0.9700	C28—O5	1.415 (3)
C9—H9B	0.9700	C28—H28A	0.9600
C10—O1	1.390 (3)	C28—H28B	0.9600
C10—H10A	0.9600	C28—H28C	0.9600
C10—H10B	0.9600	C29—O5	1.372 (3)
C10—H10C	0.9600	C29—C30	1.378 (3)
C11—C12	1.371 (3)	C29—C34	1.389 (3)
C11—O1	1.380 (3)	C30—C31	1.381 (4)
C11—C16	1.392 (3)	C30—H30	0.9300
C12—C13	1.379 (4)	C31—C32	1.363 (4)
C12—H12	0.9300	C31—H31	0.9300
C13—C14	1.365 (4)	C32—C33	1.397 (3)
C13—H13	0.9300	C32—H32	0.9300
C14—C15	1.391 (3)	C33—C34	1.393 (3)
C14—H14	0.9300	C33—C35	1.452 (3)
C15—C16	1.396 (3)	C34—O6	1.353 (3)
C15—C17	1.447 (3)	C35—N3	1.270 (3)
C16—O2	1.356 (3)	C35—H35	0.9300
C17—N1	1.275 (3)	C36—N3	1.468 (3)
C17—H17	0.9300	C36—C37	1.525 (3)
C18—N1	1.454 (3)	C36—H36A	0.9700
C18—C37	1.534 (3)	C36—H36B	0.9700
C18—H18A	0.9700	O2—H2	0.8200
C18—H18B	0.9700	O4—H4	0.8200
C19—O8	1.432 (3)	O6—H6	0.8200
C19—H19A	0.9600	O7—H7	0.8200
			
O3—C1—H1A	109.5	C20—C21—C22	121.1 (3)
O3—C1—H1B	109.5	C20—C21—H21	119.5
H1A—C1—H1B	109.5	C22—C21—H21	119.5
O3—C1—H1C	109.5	C23—C22—C21	119.4 (3)
H1A—C1—H1C	109.5	C23—C22—H22	120.3
H1B—C1—H1C	109.5	C21—C22—H22	120.3
O3—C2—C3	125.3 (3)	C22—C23—C24	121.1 (3)
O3—C2—C7	114.8 (2)	C22—C23—H23	119.5
C3—C2—C7	119.8 (3)	C24—C23—H23	119.4
C2—C3—C4	120.6 (3)	C25—C24—C23	118.9 (2)
C2—C3—H3	119.7	C25—C24—C26	121.4 (2)
C4—C3—H3	119.7	C23—C24—C26	119.8 (2)
C5—C4—C3	120.3 (3)	O7—C25—C24	121.4 (2)
C5—C4—H4A	119.8	O7—C25—C20	118.3 (3)
C3—C4—H4A	119.8	C24—C25—C20	120.2 (3)
C4—C5—C6	120.4 (3)	N2—C26—C24	123.1 (2)
C4—C5—H5	119.8	N2—C26—H26	118.4
C6—C5—H5	119.8	C24—C26—H26	118.4
C7—C6—C5	119.1 (2)	N2—C27—C37	112.89 (19)
C7—C6—C8	120.2 (2)	N2—C27—H27A	109.0
C5—C6—C8	120.7 (2)	C37—C27—H27A	109.0
O4—C7—C6	122.0 (2)	N2—C27—H27B	109.0
O4—C7—C2	118.3 (2)	C37—C27—H27B	109.0
C6—C7—C2	119.6 (2)	H27A—C27—H27B	107.8
N4—C8—C6	122.1 (2)	O5—C28—H28A	109.5
N4—C8—H8	118.9	O5—C28—H28B	109.5
C6—C8—H8	118.9	H28A—C28—H28B	109.5
N4—C9—C37	111.28 (19)	O5—C28—H28C	109.5
N4—C9—H9A	109.4	H28A—C28—H28C	109.5
C37—C9—H9A	109.4	H28B—C28—H28C	109.5
N4—C9—H9B	109.4	O5—C29—C30	124.4 (3)
C37—C9—H9B	109.4	O5—C29—C34	115.6 (2)
H9A—C9—H9B	108.0	C30—C29—C34	120.0 (3)
O1—C10—H10A	109.5	C29—C30—C31	119.8 (3)
O1—C10—H10B	109.5	C29—C30—H30	120.1
H10A—C10—H10B	109.5	C31—C30—H30	120.1
O1—C10—H10C	109.5	C32—C31—C30	120.9 (3)
H10A—C10—H10C	109.5	C32—C31—H31	119.5
H10B—C10—H10C	109.5	C30—C31—H31	119.5
C12—C11—O1	125.0 (3)	C31—C32—C33	120.2 (3)
C12—C11—C16	120.1 (3)	C31—C32—H32	119.9
O1—C11—C16	114.9 (2)	C33—C32—H32	119.9
C11—C12—C13	120.2 (3)	C34—C33—C32	119.1 (3)
C11—C12—H12	119.9	C34—C33—C35	121.7 (2)
C13—C12—H12	119.9	C32—C33—C35	119.2 (2)
C14—C13—C12	120.3 (3)	O6—C34—C29	118.8 (2)
C14—C13—H13	119.9	O6—C34—C33	121.2 (2)
C12—C13—H13	119.9	C29—C34—C33	120.0 (2)
C13—C14—C15	120.8 (3)	N3—C35—C33	123.2 (2)
C13—C14—H14	119.6	N3—C35—H35	118.4
C15—C14—H14	119.6	C33—C35—H35	118.4
C14—C15—C16	118.7 (2)	N3—C36—C37	113.1 (2)
C14—C15—C17	120.3 (2)	N3—C36—H36A	109.0
C16—C15—C17	120.9 (2)	C37—C36—H36A	109.0
O2—C16—C11	118.4 (2)	N3—C36—H36B	109.0
O2—C16—C15	121.8 (2)	C37—C36—H36B	109.0
C11—C16—C15	119.8 (2)	H36A—C36—H36B	107.8
N1—C17—C15	122.3 (2)	C36—C37—C18	109.1 (2)
N1—C17—H17	118.8	C36—C37—C27	111.42 (19)
C15—C17—H17	118.8	C18—C37—C27	107.39 (18)
N1—C18—C37	111.7 (2)	C36—C37—C9	108.6 (2)
N1—C18—H18A	109.3	C18—C37—C9	110.63 (19)
C37—C18—H18A	109.3	C27—C37—C9	109.64 (19)
N1—C18—H18B	109.3	C17—N1—C18	119.5 (2)
C37—C18—H18B	109.3	C26—N2—C27	116.9 (2)
H18A—C18—H18B	107.9	C35—N3—C36	117.2 (2)
O8—C19—H19A	109.5	C8—N4—C9	120.7 (2)
O8—C19—H19B	109.5	C11—O1—C10	118.6 (2)
H19A—C19—H19B	109.5	C16—O2—H2	109.5
O8—C19—H19C	109.5	C2—O3—C1	118.8 (2)
H19A—C19—H19C	109.5	C7—O4—H4	109.5
H19B—C19—H19C	109.5	C29—O5—C28	118.0 (2)
O8—C20—C21	125.5 (3)	C34—O6—H6	109.5
O8—C20—C25	115.1 (3)	C25—O7—H7	109.5
C21—C20—C25	119.3 (3)	C20—O8—C19	117.6 (3)

### Hydrogen-bond geometry (Å, °)

**Table:**

*D*—H···*A*	*D*—H	H···*A*	*D*···*A*	*D*—H···*A*
O2—H2···N1	0.82	1.87	2.592 (3)	147
O4—H4···N4	0.82	1.84	2.567 (2)	147
O6—H6···N3	0.82	1.89	2.623 (3)	148
O7—H7···N2	0.82	1.88	2.612 (3)	148
C27—H27B···N1	0.97	2.56	2.936 (3)	103
C26—H26···O5^i^	0.93	2.58	3.309 (3)	136
C28—H28C···O2^i^	0.96	2.57	3.387 (3)	143
C12—H12···O6^ii^	0.93	2.59	3.400 (3)	146
C32—H32···O3^iii^	0.93	2.42	3.289 (3)	155
C36—H36B···O8^iv^	0.97	2.55	3.399 (3)	146

Symmetry codes: (i) −*x*, −*y*+2, −*z*; (ii) −*x*−1, −*y*+2, −*z*; (iii) *x*+1, *y*, *z*; (iv) −*x*, −*y*+2, −*z*+1.
